# Highly stable integration of graphene Hall sensors on a microfluidic platform for magnetic sensing in whole blood

**DOI:** 10.1038/s41378-023-00530-2

**Published:** 2023-05-31

**Authors:** Nishal Shah, Vasant Iyer, Zhiping Zhang, Zhaoli Gao, Juhwan Park, Venkata Yelleswarapu, Firooz Aflatouni, A. T. Charlie Johnson, David Issadore

**Affiliations:** 1 Department of Bioengineering, University of Pennsylvania, Philadelphia, PA 19104 USA; 2 Department of Electrical and Systems Engineering, University of Pennsylvania, Philadelphia, PA 19104 USA; 3 Department of Biomedical Engineering, Chinese University of Hong Kong, Shatin, Hong Kong; 4 Department of Physics and Astronomy, University of Pennsylvania, Philadelphia, PA 19104 USA; 5 Department of Chemical and Biomolecular, University of Pennsylvania, Philadelphia, PA 19104 USA

**Keywords:** Electrical and electronic engineering, Biosensors

## Abstract

The detection and analysis of rare cells in complex media such as blood is increasingly important in biomedical research and clinical diagnostics. Micro-Hall detectors (μHD) for magnetic detection in blood have previously demonstrated ultrahigh sensitivity to rare cells. This sensitivity originates from the minimal magnetic background in blood, obviating cumbersome and detrimental sample preparation. However, the translation of this technology to clinical applications has been limited by inherently low throughput (<1 mL/h), susceptibility to clogging, and incompatibility with commercial CMOS foundry processing. To help overcome these challenges, we have developed CMOS-compatible graphene Hall sensors for integration with PDMS microfluidics for magnetic sensing in blood. We demonstrate that these graphene μHDs can match the performance of the best published μHDs, can be passivated for robust use with whole blood, and can be integrated with microfluidics and sensing electronics for in-flow detection of magnetic beads. We show a proof-of-concept validation of our system on a silicon substrate and detect magnetic agarose beads, as a model for cells, demonstrating promise for future integration in clinical applications with a custom CMOS chip.

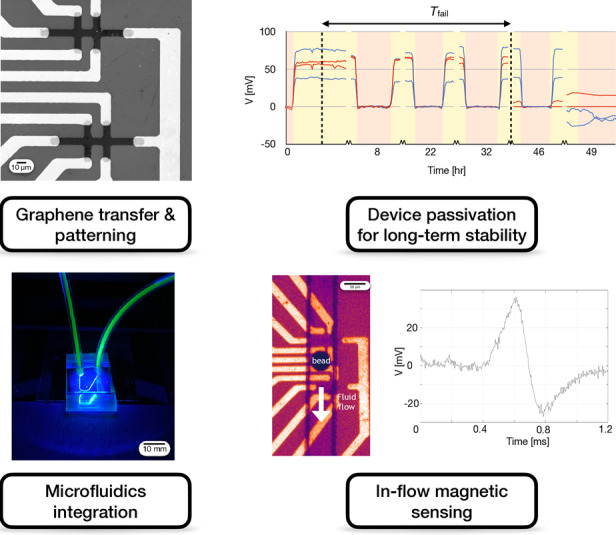

## Introduction

The detection of rare cells (<10 cells/mL), such as circulating tumor cells (CTCs) and pathogens, in clinically accessible liquid biopsies such as blood or sputum offers enormous potential for disease diagnostics^1–6^. One particularly successful strategy for rare cell sensing has been to immunomagnetically label targeted cells with antibody-functionalized superparamagnetic nanoparticles (MNPs) and then detect these labeled cells via their demagnetization field by flowing them serially over micrometer-scale magnetic field sensors. This approach shows two key strengths: 1. The negligible magnetic susceptibility of biological materials (e.g., blood, sputum, urine) obviates sample preparation steps, reducing the loss of rare cells and simplifying the clinical workflow; and 2. Magnetic sensors can be miniaturized to the micro- and nanometer scale and integrated with supporting electronics without the need for bulky supporting optics or instrumentation. Moreover, because micromagnetic sensors can be scaled to match the size of the cells that they are detecting, these cells can be measured individually such that rare cells can be identified among a vast background of unbound MNPs and nonspecifically labeled cells. Microfluidic-based magnetic separation devices^6–8^ and sensors based on the giant magnetoresistance (GMR) effect^9–13^, magnetic susceptometry^14–16^, nuclear magnetic resonance (NMR)^17–19^, and the Hall effect^20–24^ have all been developed for the detection of targeted molecules or cells and these techniques have also shown several promising results in preclinical testing.

Micro-Hall detectors (µHDs), in particular, have demonstrated promise for rare cell detection due to their excellent sensitivity and linear response to magnetic fields. µHDs can be more easily scaled to single-cell volumes than techniques such as NMR, which suffer from low signal-to-noise ratio (SNR) when applied in cell-sized volumes^17–19^. The small measurement volumes of µHDs, matched to the size of a single cell, are essential to reduce the impact of background signal from MNPs unbound to cells. Unlike GMR sensors, which are designed to operate with extremely high sensitivity within a narrow dynamic range^11^, µHDs can operate with a linear magnetic response even in the large fields (>0.1 T) that are typically required to fully magnetize the MNP labels^25^. The ability to fully magnetize MNPs using large applied fields enhances the detected signal compared to a partially magnetized scenario. However, the utility of this technology has been limited because the high sensitivity of μHDs to rare cells relies on serially interrogating each cell in a sample. The throughput of these sensors is crucial because of the large sample volumes that must be analyzed to identify rare cells. Moreover, the sensor dimensions must approximate those of the cell to ensure that each cell passes consistently through its sensor’s region of detection, but the requisite microscale channels are susceptible to clogging by unprocessed whole blood samples. A promising solution to the challenges of realizing clinically practical μHDs is to fabricate vast arrays of μHDs such that multiple streams of cells can be inspected in parallel and the clogging of any one channel does not stop overall device operation. The use of parallelization to increase the throughput of fundamentally slow microfluidic processes has been successful in recent years in a wide range of applications, including immunomagnetic sorting of cells and extracellular vesicles^26–30^, microparticle generation^31–35^, and digital droplet assays^36,37^. However, because each μHD must be sampled hundreds of thousands of times per second to detect cells in the flow stream^23^, these sensor arrays necessitate the on-chip logic, triggering, multiplexing, and analog-to-digital conversion that silicon complementary metal-oxide-semiconductor (CMOS) chips offer. Although there has been much excellent work integrating magnetic sensors with CMOS technology, there remain several unmet challenges specific to magnetic detection^15,38–41^. Conventional CMOS Hall sensors are implemented on the active silicon layer, which lies several microns beneath the chip surface; this distance reduces sensitivity to passing cells because the stray magnetic field of the labeled cells falls off as 1/*d*^3^, where *d* is the distance from the center of the cell to the μHD^23,42^. Furthermore, the sensitivity of silicon sensors is significantly less than that of GMR sensors or Hall sensors based on pseudomorphic high electron mobility transistor (PHEMT) technology made from two-dimensional electron gas (2DEG) structures^23,43^ (Table 1). However, these better-performing sensors are difficult to integrate with conventional CMOS.

**Table 1:** Comparison of best reported Hall sensors

Material	S_I_ (VA^−1^T^−1^)	Bias current (mA)	S_A_ (mVT^−1^)	B_min_ (nTHz^-0.5^)	Frequency (kHz)	μ (cm^2^V^−1^s^−1^)
Bi^54–56,83^	0.3–0.5	4.5, 4	2	550	1	18,000
Si^57,58^	80	0.5	40	~1000	277	900
InSb^59,60^	140	0.1	14	77	NR	17,453
*CVD Graphene* + *Passivation (Shah et al.)*	484	1	484	4000	3	9500
Graphene h-BN^84^	5700	~0.05^a^	300	50	3	
InAs/GaSb^43^	357	0.1	35.7	500	3	30,000
GaAs/AlGaAs III-V^58^	1100	0.5	550	~1100	277	7700
MoS_2_^61^	2996	0.0006	1.7976	NR	NR	33.4

S_I_ is the current-related sensitivity, S_A_ is the absolute sensitivity, *B*_*min*_ is the minimum detectable magnetic field and μ is the carrier mobility

^a^Calculated from absolute sensitivity since it was not reported

Two-dimensional materials (2DMs), a family of atomically thin materials exhibiting a wide range of exotic mechanical and electronic properties, have recently emerged as a promising solution for achieving high sensing performance while simplifying CMOS integration. Since 2DMs can be released from their growth substrates and transferred onto the top of CMOS chips, these materials can be integrated without requiring processes such as wafer bonding or high-temperature annealing, which are expensive and potentially damaging to sensitive electronics^44^. Several examples of 2DM-CMOS integrated chips have been realized in recent years to leverage these advantages for broadband imaging^45^ and gas sensing applications^46^.

Within the 2DM family, graphene is a particularly attractive candidate for Hall sensing due to relatively mature wafer-scale synthesis and transfer techniques^47–49^, as well as its extremely high room-temperature carrier mobility^50–52^. Graphene μHDs have been shown to outperform state-of-the-art Hall sensors made from semiconductor materials such as bismuth^53–56^, silicon^57,58^, gallium arsenide (GaAs)^58,59^, and indium antimonide (InSb)^59,60^, as well as emerging 2DMs such as molybdenum sulfide^61^ (Table 1). Additionally, the graphene carrier density, and thus the magnetic sensitivity, can be tuned by varying the backgate voltage applied to the substrate relative to the graphene surface, providing an in situ method of tuning the sensor’s performance^52^. Graphene μHDs have previously been integrated with CMOS in hybrid systems, wherein the graphene is placed on top of an integrated circuit (IC) and is electrically connected to biasing and readout electronics by wire-bonding to an external circuit board^40,62^. However, to the best of our knowledge, graphene Hall sensors have not yet been combined with CMOS technology for the purpose of in-flow magnetic sensing, despite the compelling advantages of such a combined system.

In particular, the stability of the microelectronic components associated with graphene Hall sensors exposed to ionic fluids and biofouling is a major challenge. The optimal tradeoff lies in maximizing the thickness of the sensor passivation layer while minimizing the distance between the sensor and the target. Under ambient conditions, the stability of graphene Hall sensors encapsulated in hexagonal boron nitride (hBN) has been confirmed out to 190 days^63^. Graphene Hall sensors have been integrated with CMOS ICs with 400 nm of PMMA passivation, although their stability in biologically complex fluids has not been measured^62^. Despite the immense interest in graphene Hall sensors, their adoption has been limited by the constraints of these strict tradeoffs between magnetic sensitivity and biological stability.

**Fig. 1:**
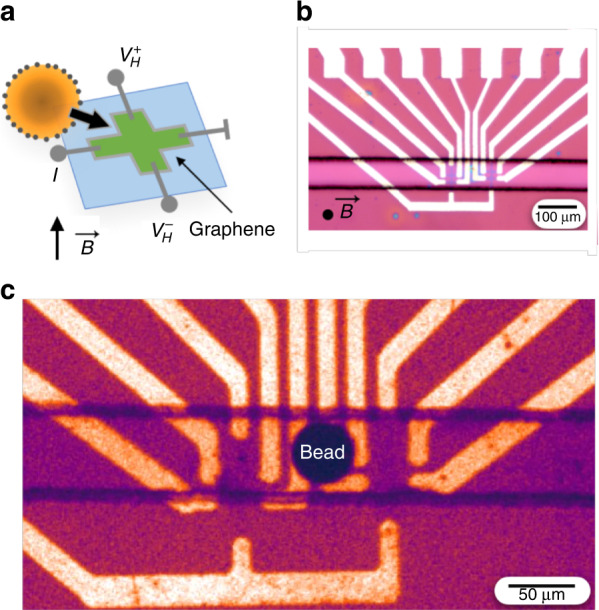
Developing microfluidic graphene Hall sensors (μGS) for rare cell detection. **a** Schematic of a cell labeled with ligand-specific nanoparticles traveling over a single μGS with a perpendicularly applied magnetic field. **b** Micrograph of the PDMS microfluidic channel covering the sensor region. The applied magnetic field is indicated as coming out of the page. **c** Micrograph of a magnetic agarose bead passing over the sensors

To evaluate graphene as an effective material for high-sensitivity CMOS-compatible magnetic sensors, we have developed a fabrication strategy that enables a graphene Hall sensor (μGS) to be combined with microelectronic and microfluidic components to detect passing magnetic beads in complex biofluids (Fig. 1a). Importantly, we have developed fabrication strategies that allow the graphene sensor output to detect the magnetic field of passing beads over multiple hours without being sensitive to complex backgrounds such as whole blood. We first grow graphene using chemical vapor deposition (CVD), transfer it to a silicon chip, and photolithographically pattern the μGS array. We encapsulate the μGS sensor array with a passivation layer to protect the graphene from biofluids for long-term stable sensing in whole blood. Subsequently, we align and irreversibly bond a PDMS chip with soft lithographically defined microfluidic channels (Fig. 1b) to the silicon chip using oxygen plasma activation (Fig. 1c). We demonstrate the stability of our hybrid microfluidic-microelectronic system out to 39 h of continuous operation in human blood. We characterize the sensing performance of our device in bovine serum albumin (BSA) and whole blood using magnetic agarose beads as a model system for detecting magnetic particles in biologically complex fluids. We use microbeads as a model for cells because they can offer a controlled magnetic signal, allowing the technology to be independently evaluated without the results being confounded by cell-to-cell variability of immunomagnetically labeled cells^23,64–66^. The proof-of-concept results reported here lay the foundation for graphene Hall elements to be incorporated into CMOS ICs through post-fabrication and applied to rare cell assays.

## Results

### Device fabrication

**Fig. 2:**
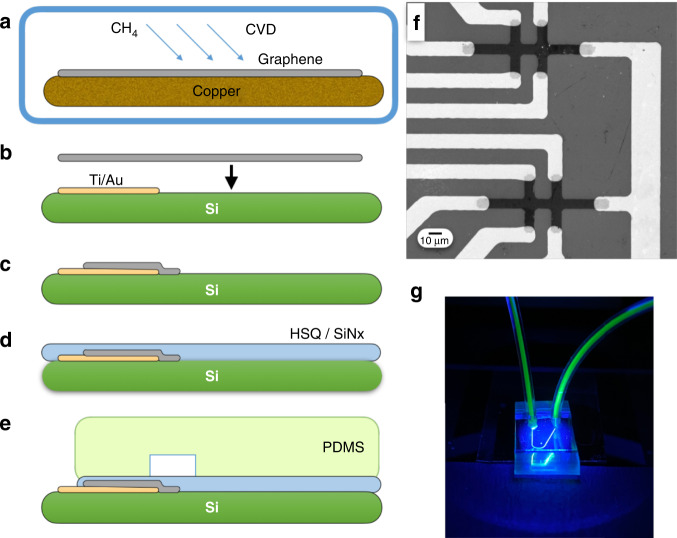
μGS device fabrication. **a** Graphene is grown on copper foil through chemical vapor deposition (CVD). **b** The graphene sheet is transferred onto a silicon chip with Ti/Au electrodes. **c** The graphene is patterned using photolithography to create μGSs. **d** The μGSs are passivated with a layer of HSQ and then with a layer of Si_3_N_4_ through CVD. **e** PDMS microfluidic channels are plasma bonded onto the passivation layer. **f** SEM image of the patterned graphene in contact with the metal electrodes. **g** A photograph of the device under blue light showing the microfluidic channel and tubing

Graphene is grown via a previously published low-pressure chemical vapor deposition (CVD) process^67^. During growth, 10 sccm CH_4_ + 80 sccm H_2_ is flowed over a copper foil (Alfa Aesar Item 46365) at 1020 °C for 20 min in a chamber pumped to 50 mTorr (Fig. 2a). The as-grown monolayer graphene is transferred using the bubble transfer method^67^ onto a chip with prefabricated Au electrodes (Fig. 2b) that is prepared on a silicon wafer with a 280 nm layer of thermally grown SiO_2_ (University Wafer 3333). The electrodes are lithographically defined using lift-off and electron beam evaporation (2 nm Ti, 40 nm Au) (Lesker PVD). The graphene sheet is lithographically patterned using positive-resist photolithography followed by oxygen plasma etching at 50 W for 30 s (Fig. 2c). The length of the double-cross Hall bar was 70 μm, the width of the Hall bar was 28 μm, and the width of each arm was 8 μm. An SEM image of the graphene sensors confirmed the presence of an intact graphene layer atop the electrodes (Fig. 2f). After patterning, the graphene is annealed in H_2_ (250 sccm)/Ar (1000 sccm) at 225 °C in a quartz tube for 1 h to eliminate photoresist residues^68,69^. After annealing, the graphene is encapsulated by first spin-coating 300 nm of hydrogen silsesquioxane (HSQ) (XR-1541, Dow Corning) and then depositing 140 nm of silicon nitride (Si_3_N_4_) (Fig. 2d) via chemical vapor deposition (Oxford PlasmaLab 100). Encapsulation layer thicknesses were measured using ellipsometry (Filmetrics F40).

Microfluidic channels are fabricated using standard soft lithography techniques and integrated directly on top of the μGS chip. The microfluidic channel is 50 μm wide and 50 μm tall with one inlet and one outlet. The mold for the channel was fabricated on a silicon wafer using UV photolithography (SU8 2050, MicroChem). The PDMS was prepared in a 1:10 ratio of curing agent to elastomer and baked at 65 °C for 2 h. The passivated μGS chip and the PDMS piece are activated using a barrel asher (Anatech) with an O_2_ plasma at 50 W for 30 s to activate their surfaces for bonding. The two pieces were bonded together using a mask aligner to align the microfluidic channels and the μGS (Fig. 2e). The alignment of the PDMS device to the silicon substrate was not affected by PDMS shrinkage, which can range from 1.06–1.67% under our PDMS curing conditions^70,71^. Because our channel width was designed to be 50 μm, the small changes in channel width due to shrinkage do not noticeably affect our ability to align our μGS sensor array (width = 33 μm) into the center of the channel. Fluidic tubing for the input and output is connected to the PDMS, and flow was driven using positive pressure (Fig. 2g). To minimize the loss of beads and clogging due to magnetophoretic forces from the edge of the permanent magnet, we used a 0.25-inch diameter NdFeB magnet (K&J Magnetics D4C-N52) that was positioned directly beneath the sensing region of the chip.

### Graphene characterization

**Fig. 3:**
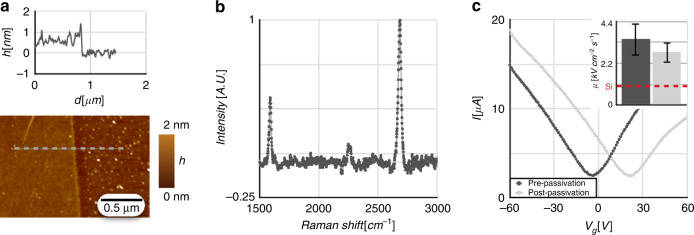
Characterization of graphene in the μGS. **a** Graphene sheet thickness measured by atomic force microscopy. **b** Unique spectral peaks of graphene observed using Raman spectroscopy. **c** Current-gate voltage response in a characteristic device showing the change in carrier mobility before and after passivation. The Dirac point shifts due to the change in surface interactions of the μGS. The inset compares mobility before and after passivation to that of silicon sensors

We first evaluated the quality of the CVD-grown graphene. The thickness of the graphene layer was measured using AFM to be ∼0.6 nm (Fig. 3a), consistent with other reports of monolayer graphene^72,73^. Measurements from Raman spectroscopy using a 532 nm laser showed peaks at 1580 cm^−1^ and at 2690 cm^−1^; these peaks (known as the G peak and the 2D peak, respectively) are characteristic of graphene. The Raman spectrum indicates the single-layer quality of the graphene since defects in the sheet broaden the G and 2D peaks while contributing an additional D peak at 1350 cm^−1^. The 2D-G peak ratio provides information about the number of layers, typically exceeding 2 for monolayer graphene and decreasing with additional layers. We observed no D peak at 1350 cm^−1^, as well as a high 2D-G peak ratio of 2.20 and a narrow 2D FWHM (full width at half-maximum) of 29.78 cm^−1^, confirming the quality of the monolayer graphene synthesis (Fig. 3b)^74^. The average electron mobility from sixty-seven passivated μGSs was measured to be *μ* = 4600 ± 300  cm^2^V^−1^s^−1^ (Fig. 3c) by the direct transconductance method^75^, which calculates electron mobility *μ* through the dependence of the transconductance *g*_*m*_ on the backgate voltage:

1 $$gm = \frac{{\partial I}}{{\partial V_G}} = \mu C_{ox}\frac{W}{L}V_{DS} \Rightarrow \mu = \frac{{g_mL}}{{C_{ox}WV_{DS}}}$$

In this equation, *C*_*ox*_ is the per-unit-area gate capacitance; *V*_*DS*_ is the constant voltage bias supplying the drain current *I;* and *L* and *W* represent the sheet dimensions along and transverse to the drain current, respectively. The electron mobility of the μGS decreased by ∼30% after passivation, although it remained considerably higher than that of silicon sensors (Fig. 3c, inset). The mobility calculated using this method falls in the same range as previously studied graphene Hall sensors (Table 1).

**Fig. 4:**
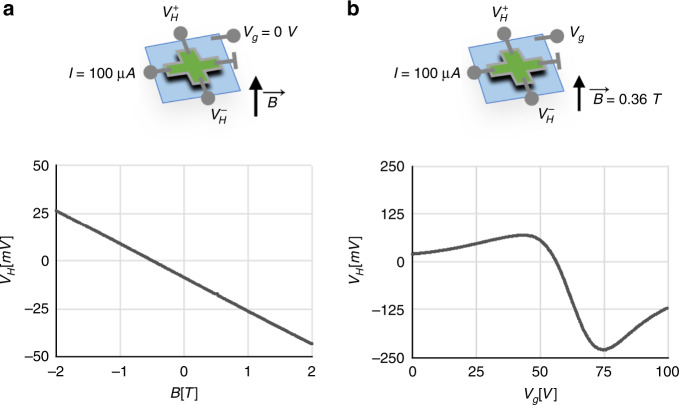
Magnetic response of the graphene μHD sensors. **a** The linear response of the Hall voltage to magnetic field strength gives a current-related sensitivity of 175 VA^−1^T^−1^. **b** Hall voltage response to back-gate voltage shows the ambipolarity of graphene. The measurement field is chosen to be |B| = 0.36 T to match the magnetizing field used for the in-flow experiments

We next characterized the magnetic sensing performance of µGS sensors in dry conditions. The linear relationship between the Hall voltage, *V*_*H*_, and field strength, $\overrightarrow B$, is shown in Fig. 4a and the absolute sensitivity, measured as the slope of the curve, was measured to be 175 mVT^−1^ (R^2^ = 0.99997) (Quantum Design, Physical Characterization System). Additionally, the linear relationship was consistent over a dynamic range of $\overrightarrow B$ from −2 T to 2 T. Biasing at a current of 100 μA, we calculated the current-related sensitivity, S_I_ = 175 VA^−1^T^−1^, which is similar to reported values for CMOS-compatible graphene Hall sensors^40^. The tunability of the Hall voltage as a function of the back-gate voltage, *V*_*g*_, (Fig. 4b) confirmed the ambipolarity of graphene and the change in charge carriers from holes to electrons. The largest *V*_*H*_ response of −229 mV was recorded at *V*_*g*_ = 74 V. The gate voltages could be shifted to lower values by replacing the 280 nm SiO_2_ layer with a thinner layer of a material with a larger dielectric constant, such as Al_2_O_3_ or HfO_2_^76^. The noise within the relevant bandwidth for the μGS (100 Hz to 100 kHz) was measured by an FFT spectrum analyzer (Stanford Research SR770). The minimum detectable field *B*_*min*_ = 1.35 μTHz^-0.5^ was measured at the typical signal frequency of 3 kHz and a bias voltage of *V*_*bias*_ = 10.3 V (Supplementary Fig. S1). Under these bias conditions, the measured 1/*f* characteristic extends to 100 kHz, where the noise is ~200 nTHz^-0.5^; therefore, CMOS-integrated implementations of these sensors could use lock-in detection to achieve a better noise floor by shifting the signal content to higher frequencies^58,77^. This lock-in technique would ultimately be limited by the sensor thermal noise floor, which can be estimated to first order by considering the Johnson noise associated with the μGS resistance (*R*_μ*GS*_ = 3.6 *k*Ω). Assuming an operating temperature of 300 K and *I*_*bias*_ = 3 mA  and using measured values for *S*_*I*_:

2 $$B_{{\min},th}\approx \frac{{\sqrt{4kTR}}_{\mu GS}}{S_{I}I_{bias}}=12\frac{{\rm{nT}}}{\sqrt {\rm{Hz}}}$$

By extrapolating the measured 1/f noise, the 1/f noise corner (the frequency where thermal and flicker noise are equally prevalent) is at ~220 MHz. Beyond this frequency, we would expect lock-in techniques to provide little additional benefit.

### Measurement of device stability and sensitivity in complex biofluids

**Fig. 5:**
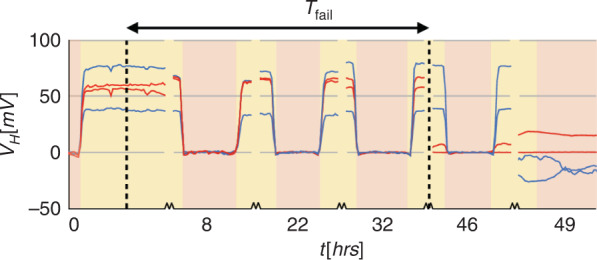
Stability of graphene μGS devices in blood plasma. Two of the four HSQ-Si_3_N_4_ encapsulated devices (indicated in red) fail after 32 h of exposure to plasma and the other two (indicated in blue) fail after 46 h, giving an average T_fail_ of 39 h. The yellow background denotes when the external magnetic field is applied and the pink background denotes when the external magnetic field is removed

We characterized the efficacy of our encapsulation method by quantifying the stability of the μGS magnetic field response when the sensor was placed in contact with blood plasma. We used blood plasma as a medium for the stability measurements to capture the ionic properties of blood without concern for cell sedimentation in a nonflowing system. We compared our various passivation approaches and measured the average time for the sensors to fail, *T*_*fail*_, for each method. *T*_*fail*_ was quantified as the duration of time the sensor contacts blood plasma until the magnetic field response drops by more than 90%. The use of a bilayer of high-temperature HSQ and Si_3_N_4_ achieved a *T*_*fail*_ = 39 h (Fig. 5). This combination was multiple orders of magnitude better than the other encapsulation strategies that failed almost immediately (Table 2). The nonpassivated device along with the single-layer SiO_2_ — HSQ-only and PVD-SiO_2_ — devices all failed in <1 min. Of the photoresist-passivated devices, the PMMA device failed after 31 ± 8 s, while the SU8 device failed after 2 ± 0 min. Of the devices that had two separate deposition layers, the PVD-SiO_2_/Si_3_N_4_ device failed after 92 ± 45 s, while the low-temp HSQ/low-temp Si_3_N_4_ device failed after 4 ± 0 h and the high-temp HSQ/high-temp Si_3_N_4_ device failed after 39 ± 4 h. The failure variability is measured as the standard deviation of the *T*_*fail*_ recorded across all four sensors on a single chip.

**Table 2:** Comparison of stability of various passivation methods

Passivation method	No passivation	HSQ Only	PVD SiO_2_	PVD SiO_2_ /Si_3_N_4_	PMMA	SU8	Low temp. HSQ/Si_3_N_4_	High temp. HSQ/Si_3_N_4_
Thickness (nm)	N/A	300	50	600	200	1000	650	440
*T*_fail_	<1 min	<1 min	<1 min	<10 min	<10 min	<10 min	4 h	39 h

The initial Hall measurement (*t* = 0, prior to introducing fluid) reveals that the four tested sensors (all fabricated on the same chip) exhibit variability in their Hall responsivity. Such variability is a common challenge encountered with sensors and devices based on two-dimensional materials. While not completely understood, its sources are typically attributed to chemical doping, mechanical strain, and lattice defects introduced during the synthesis and device fabrication process^78^. The variability is known to be static (i.e. time-invariant) in nature; thus, it can be compensated by adjusting the bias of each sensor or using back-gating terminals to tune the carrier density of each device^50^. In these experiments, we focused on quantifying the relative performance change of each device over time to assess whether passivation effectively protects the sensor from the degrading effects of fluid contamination.

**Fig. 6:**
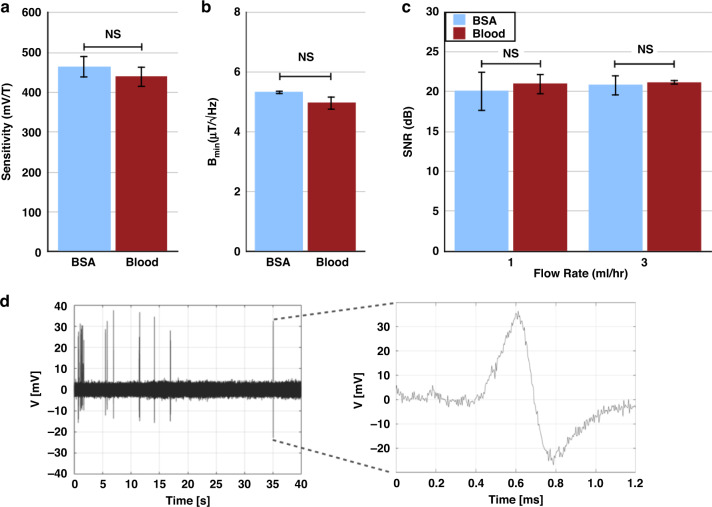
Characterization of graphene μGS performance in complex media. Absolute sensitivity measurements between BSA and blood are not significantly different (two-tailed Student’s *t* test; *p* = 0.076). **b** *B*_*min*_ measurements between BSA and blood are not significantly different (two-tailed Student’s *t* test; *p* = 0.32). **c** SNR measurements of magnetic agarose beads between BSA and blood are not significantly different at 1 mL/h and 3 mL/h (two-tailed Student’s *t* test; *p* = 0.74, *p* = 0.77). **d** Bead signals over the course of 40 s and a zoom-in of the *V*_*H*_ from one representative bead

After confirming the long-term stability of our device, we tested the μGS magnetic sensitivity in complex biofluids. We first tested the DC Hall response while flowing 1% BSA and 25% diluted blood. We used 25% diluted blood due to irreversible channel clogging from cell aggregates in whole blood (45% Ht) and 50% blood at our operating flow rates. At 25% diluted blood, channel clogging was not noticed in any of our experiment runs. Dilution was preferred to lysis buffers and filtering to minimize lossy sample preparation steps^79–81^. The difference between the absolute sensitivities at 124.5 mVT^−1^ and 123.5 mVT^−1^ for BSA and diluted blood, respectively, was not statistically significant (two-tailed Student’s *t* test; *p* = 0.91) (Supplementary Fig. S2). To improve the sensitivity, we performed a backgate sweep by measuring *V*_*H*_ as a function of *V*_*g*_ applied to the silicon substrate. After identifying the *V*_*g*_ bias where *V*_*H*_ shows a maximum (17 V), we measured the backgate-tuned sensitivity of the μGS to be 464.5 mVT^−1^ and 439.8 mVT^−1^ in BSA and blood, respectively, which is higher than the reported sensitivity of CMOS-graphene chips and 2DEG-based Hall sensors. Additionally, the difference between the absolute sensitivities for BSA and diluted blood was not statistically significant (two-tailed Student’s *t* test; *p* = 0.076) (Fig. 6a). We also measured the noise spectra of the device while flowing BSA and diluted blood and calculated the *B*_*min*_ at the relevant bandwidth of 3 kHz to be 5.12 μTHz^-0.5^ and 4.99 μTHz^-0.5^, respectively. The difference between the *B*_*min*_ in the two fluids was also not statistically significant (two-tailed Student’s *t* test; *p* = 0.32) (Fig. 6b).

### In-flow detection

We initially tested the magnetic response from 50 μm diameter ferrofluid droplets within an inert, nonfouling medium. This test allows us to evaluate the capability of the magnetic sensors to detect transiently passing objects independent of any effects from a complex medium. The dispersant and continuous phases of the water-in-oil droplets were a surfactant-stabilized ferrofluid (Ferrotec EMG 705) with a nanoparticle concentration of 3.9% v/v and HFE 7500 (3M) + 2% v/v Krytox 157 FSH oil (Chemours), respectively. The droplets were generated in a flow-focusing generator to allow for even spacing between droplets (Supplementary Fig. S3)^82^. The PDMS device was composed of a single channel with a height of 50 μm and a width of 50 μm to ensure that the droplets were in plug flow.

To test the μGS’s ability to sense passing magnetic objects in flow, we first used 50 μm ferrofluid droplets suspended in oil (HFE + 2% Krytox). These droplets – with a magnetic moment of 1.71 × 10^−10^ Am^2^ that generates a V_H_ similar to that of immunomagnetically labeled cells – are designed to pass in plug flow, thus allowing the sensor’s performance to be tested independently of its position in the channel. The ferrofluid emulsions are generated in a flow-focusing generator (1 mL/h continuous phase and 0.05 mL/h dispersant phase) and then directly flowed over the sensor. The average signal duration was 217 μs and the SNR in the relevant bandwidth of 1–5 kHz was 58.5 or 17.7 dB (Supplementary Fig. S4).

To test the μGS in clinically relevant biofluids, we generated 47 μm diameter agarose (FF-AG) beads loaded with ferrofluid that could be spiked into BSA and blood and run through the device. We use 47 μm beads such that we can independently assess the performance of the sensor to detect passing magnetic materials without the signal strength being confounded by the variance of bead position in the microchannel. Moreover, the generation of smaller beads is made challenging by the high viscosity of the agarose and ferrofluid solutions that compose the dispersant phase. However, the stray field detected by the sensor from a 47 μm agarose bead matches that of a 12 μm cell labeled with 5 × 10^4^ 50-nm magnetic nanoparticles. Using BSA and whole blood as the flow media in these experiments allows for the measurements to capture the effects of ions, protein aggregates, and background cells typically found in clinical samples. The SNR at 1 mL/h in BSA was 104.5  or 20.2 dB and 121.0  or 21.0 dB in 25% diluted blood and the difference between the SNRs was also not statistically significant (two-tailed Student’s *t* test; *p* = 0.74) (Fig. 6c). The signals from FF-AG beads suspended in BSA and diluted blood passing over the μGS along with the signal from a single FF-AG bead are shown in Supplementary Fig. S5 and Fig. 6d, respectively. To test if the detected signals were a result of the demagnetization field from the passing bead, we plotted the frequency and intensity of the signals (Supplementary Fig. S6a). Since graphene is known to be optically responsive, we turned off the ambient light (Supplementary Fig. S6b) and continued to see a response from the μGS that matches the frequency and intensity from the signals in Supplementary Fig. S6a. We then turned the ambient light back on, removed the external magnet and observed that the signals disappeared (Supplementary Fig. S6c). To confirm that the signal was not coming from electrostatic charge from the beads, we turned off the ambient light and removed the external magnet, after which we continued to see no signals (Supplementary Fig. S6d).

## Discussion

We have demonstrated CMOS-compatible μGSs that can match the performance of other high-performing micro-Hall detectors. We also developed a passivation strategy for μGSs that combines a layer of spin-coated HSQ and CVD Si_3_N_4_ that allows for stable device operation in unprocessed plasma. We achieved an absolute sensitivity of 440 mVT^−1^, which was significantly higher than the corresponding values for silicon and other bulk semiconductor Hall sensors^57,83^. Our S_A_ was less than that of the best reported CVD-grown graphene, but higher than the S_A_ of other passivated graphene sensors^66^. Additionally, while the magnetic field sensitivity, *B*_*min*_, was below the best reported graphene or 2DEG Hall sensors^43,50–52,84^, our sensors can be integrated with CMOS, permitting this to be addressed by using on-chip lock-in techniques to approach the thermal noise limit^58,77^. The maximum mobility of the μGS was 9500 cm^2^V^−1^s^−1^, with an average mobility of the μGS of 4630 cm^2^V^−1^s^−1^, which was significantly higher than that of bulk semiconductor Hall sensors and on the same order as median values for 2DEG Hall sensors. Notably, our mobility is nearly 5× that of other CMOS-compatible graphene and silicon sensors, indicating a cleaner and more robust passivation process. The mobility in principle can be enhanced further by encapsulating the graphene between exfoliated hexagonal boron nitride h-BN layers^84^, which reduces the effect of charge traps on the graphene layer. However, despite recent work to batch fabricate^63^ these chips using CVD, their performance has not matched that of graphene encapsulated in exfoliated h-BN.

Our passivation strategy allowed for Hall voltage measurements out to nearly 2 days in the presence of blood plasma, indicating the robustness and reusability of the device. To be used with whole blood, the Hall sensors must be sufficiently protected from the biofluids while minimizing the distance between the target and the sensor. Our study of various passivation materials found that duplex layers comprised of spun-on amorphous SiO_2_ made of cured HSQ and capped with PECVD-grown Si_3_N_4_ performed well as a barrier to protect our devices from the complex biofluids. The other passivation layers that we experimentally evaluated, including PVD SiO_2_, HSQ, PMMA, SU-8, and PVD SiO_2_ + Si_3_N_4,_ all failed to achieve the same performance as HSQ + Si_3_N_4_. These results were consistent with prior studies that investigated microelectrode passivation failure using electron microscopy of the failed barrier layers^85^. Typical failure mechanisms include cracking due to mechanical stress, defects in the films including pinholes and particle inclusions, and diffusion and/or absorption of water and ions into the film leading to deformation and chemical reactions. Our experiments with thin organic films (PMMA and SU-8) show rapid barrier failure; these results are consistent with previous findings reporting that polymer films allow for relatively high diffusion of ions and water, leading to hydrolysis and oxidation reactions that corrode the film^86^. Although inorganic films are much more effective as barriers to water/ion diffusion, single-layer SiO_2_ films have been shown to be susceptible to water absorption when the concentration of water-binding silanol (Si-O-H) groups within the film is high. This phenomenon likely explains why our experiments with SiO_2_ films (whether through e-beam PVD or by curing HSQ) showed limited effectiveness. Furthermore, SiO_2_ deposition via e-beam PVD is quite anisotropic compared to spin-coating and conformal PECVD, which may have led to barrier breakdown near the contacts in the passivation techniques we tested involving PVD SiO_2_. A final consideration is the PECVD conditions for Si_3_N_4_ deposition, as prior work has shown that high-quality films can be achieved with less than 1 pinhole per cm^2^ for deposition temperatures above 300 °C, while much greater pinhole density was reported at lower temperatures^87^. In our findings, the duplex film with Si_3_N_4_ deposited at 150 °C provided some fluid protection (4 h) but significantly less than the duplex film with 350 °C Si_3_N_4_, suggesting that pinholes are indeed ultimately responsible for device failure.

It is important to note that, unlike in previous studies that assume no constraints on passivation thickness, composition, or deposition process, our tests sought to find a well-performing passivation strategy given the constraints set by our application. Although fabricating Hall sensors from graphene offers significant advantages in terms of sensitivity and ease of processing, their implementation does preclude the use of certain barrier layers. For example, we did not attempt to perform PECVD to deposit the initial layer of SiO_2_ since the graphene would readily etch in plasma conditions. Furthermore, the lack of reactive sites on the graphene surface makes it challenging to deposit high-quality thin oxide films on its surface using atomic layer deposition (ALD), although ongoing research into graphene-compatible ALD may resolve this issue in the near future^88^. An additional constraint exists on the use of higher-order layered films: the magnetic signal strength decays as 1/*d*^3^, where *d* is the vertical distance of a passing bead or cell over a sensor, encouraging the passivation film(s) to be as thin as possible without sacrificing device longevity. Finally, although postdeposition annealing at high temperatures (800 °C) has been shown to improve barrier performance, we were constrained from using this technique by the thermal requirements for CMOS compatibility^89^. These limitations made it impossible for us to implement passivation strategies that have been reported to protect against ionic solutions for hundreds of hours; even so, the longevity of our duplex film strategy (39 h) is more than sufficient for most particle detection applications.

Previous work integrating Hall sensors with CMOS architecture has been limited to immunoassays in microarrays^20,42^. By measuring at a constant sample flow rate of φ = 1 mL/h, we achieved throughputs on par with those in other reported magnetic flow cytometers^41,65,90^. Throughput depends on cell size and the number of labeled surface markers, but is typically limited by the shear rates of cells^91^ in microfluidic systems rather than the bandwidth of the sensor^23,40,64,65^. Thus, increasing sample throughput beyond what was demonstrated in this work requires operating several sensors in parallel. Recent advances in parallelized microfluidics have made it feasible to distribute the sample between a large number of channels (>100), which also reduces the risk of sample analysis failure due to single-channel clogging^31–35^. However, controlling a large sensor array is challenging to achieve with the planar device fabrication techniques as used in this work, where sensing and routing are implemented on the same layer. The complexity of electrical routing and external instrumentation grow proportionally more severe with the size of the array. CMOS-integrated sensors offer a practical solution to this problem, as routing and control electronics can be placed underneath the sensors to permit a scalable array. Furthermore, on-chip techniques such as matched filtering and peak detection can be used to enhance the detection SNR and compress the amount of data to be output and processed off-chip^40,65^. Our results demonstrate that μGS fabrication and passivation are feasible within the thermal constraints of CMOS compatibility (<350 °C) with standard semiconductor processing steps and equipment^89^. These results pave the way for realizing CMOS-integrated μGS arrays with a sufficient degree of parallelization for vastly increased sample throughput. The prospects of successful μGS-CMOS integration have improved in recent years due to advancements in state of the art monolithic graphene-CMOS integration for imaging^45^ and gas sensing^46^, as well as progress in wafer-scale graphene synthesis and transfer techniques^47–49^.

## Materials and methods

### Fabrication of various passivation layers

We evaluated several encapsulation methods, including thermally evaporated PVD SiO_2_, HSQ only, poly(methyl methacrylate) or PMMA, SU-8, PVD SiO_2_ + Si_3_N_4_, and HSQ + Si_3_N_4_. The thermally evaporated SiO_2_ was deposited using physical vapor deposition (Lesker PVD) to a thickness of 50 nm. In the HSQ-only device, 300 nm of XR-1541 was first spin-coated, and the chip was then soft-baked for 4 min at 80 °C and hard-baked for 15 min at 350 °C. HSQ has previously been shown to effectively encapsulate graphene devices for up to two weeks in air while enhancing mobility^92^. To investigate the suitability of the photoresists used during graphene transfer and patterning that could minimize processing steps, PMMA and SU-8 were considered as candidates. A 200 nm layer of PMMA was spin-coated and then baked for 2 min at 105 °C. A total of 1 μm of SU-8 was spin-coated in two steps, where each step involved spinning 500 nm of resist, soft-baking for 1 min at 90 °C and hard-baking for 10 min at 180 °C. Si_3_N_4_ was also considered due to its excellent passivation characteristics^93^ using plasma-enhanced chemical vapor deposition (PECVD), which allows for lower temperatures and CMOS compatibility. However, since graphene is etched away in plasma, we developed multilayer approaches for encapsulation with an initial layer of SiO_2_. The following combinations were tested: 100 nm PVD SiO_2_ + 140 nm Si_3_N_4_; low temperature 330 nm HSQ + 320 nm Si_3_N_4_; and high temperature 300 nm HSQ + 140 nm Si_3_N_4_. The first layer in the PVD SiO_2_ + Si_3_N_4_ device was deposited by electron beam evaporation to 100 nm and the Si_3_N_4_ layer was deposited to a thickness of 140 nm via PECVD at 350 °C. In the high-temperature HSQ + Si_3_N_4_ device, HSQ was deposited by spin-coating 300 nm and soft-baking at 80 °C for 4 min. The chip was then hard baked at 350 °C for 15 min, and 140 nm of Si_3_N_4_ was deposited via PECVD at 350 °C. To test a low-temperature process and determine CMOS compatibility, we fabricated an HSQ + Si_3_N_4_ device where the chip was hard baked at 150 °C for 1 h and Si_3_N_4_ was deposited at 150 °C.

### Testing device stability and sensitivity in complex media

To determine the most stable passivation method, we measured the DC Hall response from the μGS chips over time with the sensors immersed in human blood plasma. We used blood plasma rather than whole blood due to its ease of acquisition while retaining the relevant ionic concentrations of whole blood. The plasma was added to a laser-cut acrylic well that was placed directly over the μGS region and *V*_*H*_ was measured by introducing and removing a 0.5 T external magnetic field. A non-encapsulated device was used as a negative control where the graphene was exposed directly to the plasma in the fluid channel. The devices were tested with a constant source-drain voltage of 10 V, with a magnetic field of 0.36 T provided by an NdFeB magnet. We then added 100 μL of EDTA-treated human plasma onto the μGS and measured *V*_*H*_ at several timepoints to monitor device stability.

To further elucidate the magnetic performance of the μGS in the presence of biologically complex fluids such as plasma and blood, we compared the absolute sensitivity of the devices in the presence of BSA and diluted blood (1/4 dilution with BSA) by measuring the DC Hall response. Using an electromagnet (Bunting BDE-3020-12) to precisely vary the applied magnetic field from 0–38 mT, the Hall sensor response was measured with a 12.8 V battery supply while first flowing BSA and then flowing diluted blood over the sensor at 1 mL/h to determine the absolute sensitivity of the device.

### In-flow testing of ferrofluid-agarose beads

Next, to evaluate our system’s capability for detecting magnetic objects in-flow in biologically complex fluids, we used agarose beads loaded with MNPs and suspended in whole blood. The agarose beads were generated similarly to the ferrofluid droplets by generating agarose-in-oil emulsions. The agarose beads were made by first mixing low-melting agarose powder (1.5% w/v) into the ferrofluid and heating to 95 °C to fully dissolve the agarose. The aqueous ferrofluid-agarose (FF-AG) was then loaded into a syringe with the pump area heated to 60 °C using a space heater (Amazon) to keep the agarose molten. FF-AG emulsions were generated in HFE-Krytox and the output was collected in an ice bath for rapid gelation. The continuous phase was run at 5 mL/h, and the dispersant phase was run at 0.05 mL/h to generate emulsions ~50 μm in diameter. After a 1-h incubation at 4 °C, the FF-AG hydrogels were placed on a magnetic stand to remove the HFE-Krytox and washed 3 times with 1% BSA before finally resuspending in 100 μL of 1% BSA. The final concentration of the resuspended hydrogels was ~4 × 10^5^/mL and the hydrogels were measured to be 47.3 μm with a CV of 3.4%. We then added 5 μL of the FF-AG hydrogels to 1 mL of both 1% BSA and diluted blood to flow over the μGS. Each sample was then run at multiple flow rates (1 mL/h and 3 mL/h) through the μGS chip with a 1% PBS wash between each sample type. We further confirmed the magnetic response of the agarose beads by comparing the ratio of true positives to false positives for several different negative controls. These negative controls included no beads (NB), no external magnet (NM), no light source (NL), and no magnet or light (NM/NL). No-magnet and no-light controls are necessary since graphene is known to be optically and electrochemically responsive^94,95^. We measured the FF-AG counts in diluted blood at 1 ml/h as a function of threshold, measured as a multiple of the RMS_noise_. Above a threshold value of 4* RMS_noise_, no events are detected when the magnet is removed, thus confirming the magnetic response.

### Signal analysis

To analyze the output from the μGS, the signal was processed through a combination of analog and digital filters. The circuit is composed of a high-pass filter with a cutoff frequency at 1 kHz, two operational amplifiers (Texas Instruments THS4131C) in series, and a low-pass anti-aliasing filter with a cutoff frequency at 100 kHz (Supplementary Fig. S7). The output was collected using a data acquisition system (National Instruments NI-6361) at a sampling rate of 250 kHz/channel. The output was then digitally bandpass filtered (1–10 kHz) and integrated to determine the power of the signal. Using a high threshold - 10* RMS_noise_ - to prevent counting of false positives, the average SNR was between 20 dB and 23 dB, with the SNR trending higher with a higher flow rate. This can be attributed to the flicker noise decreasing at higher frequencies; as we slide the digital bandpass filter to a higher center frequency to capture faster bead events while keeping the bandwidth fixed, the integrated noise drops.

## Supplementary information

Supplementary Material
