# Supplementary material for: Highly stable integration of graphene Hall sensors on a microfluidic platform for magnetic sensing in whole blood
Source: Microsyst Nanoeng. 2023 May 31;9:71. doi: 10.1038/s41378-023-00530-2 (PMC10232500; doi:10.1038/s41378-023-00530-2)
Supplement: Supplementary file 1 — Supplementary Material [file 41378_2023_530_MOESM1_ESM.docx]

# Supplementary Information


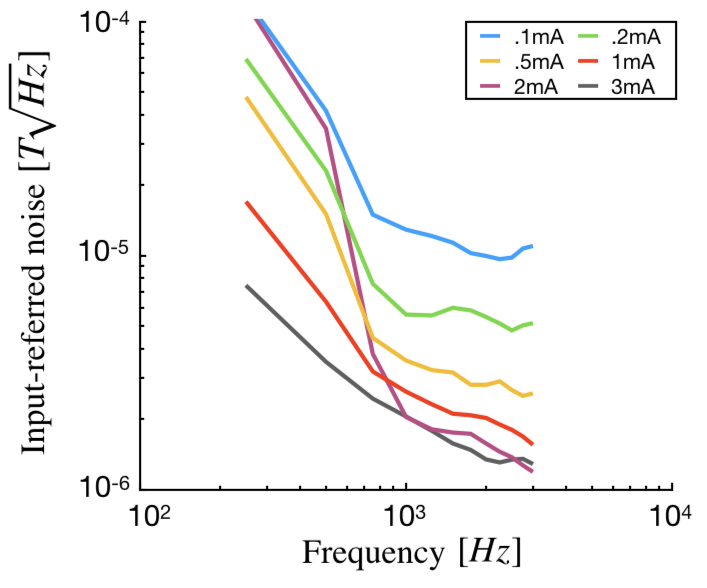


**Figure S1.** Field resolution measured in a static 0.5 T field when varying bias current.


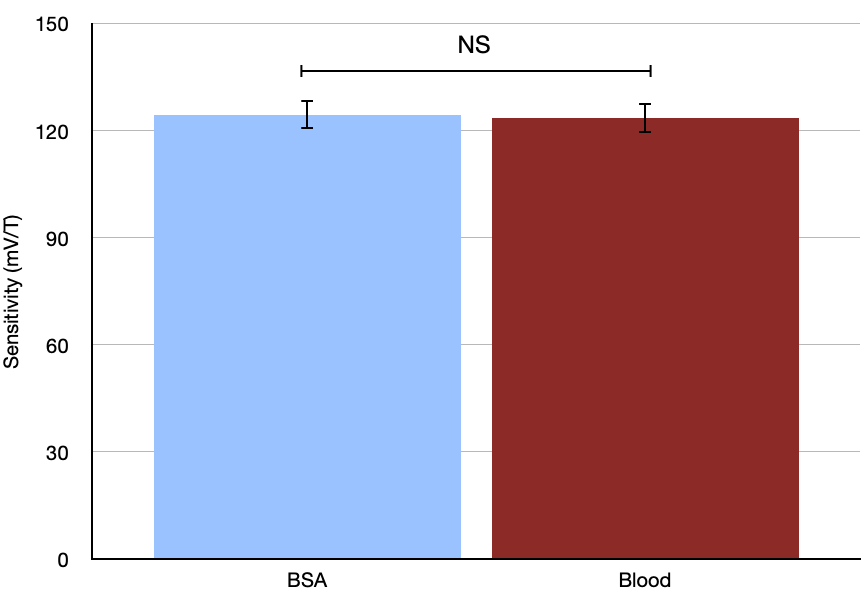


**Figure S2.** Absolute sensitivity measurements between BSA and blood on a chip without backgate tuning. The sensitivities are not significantly different (two-tailed Student’s t-test; p=0.9148).


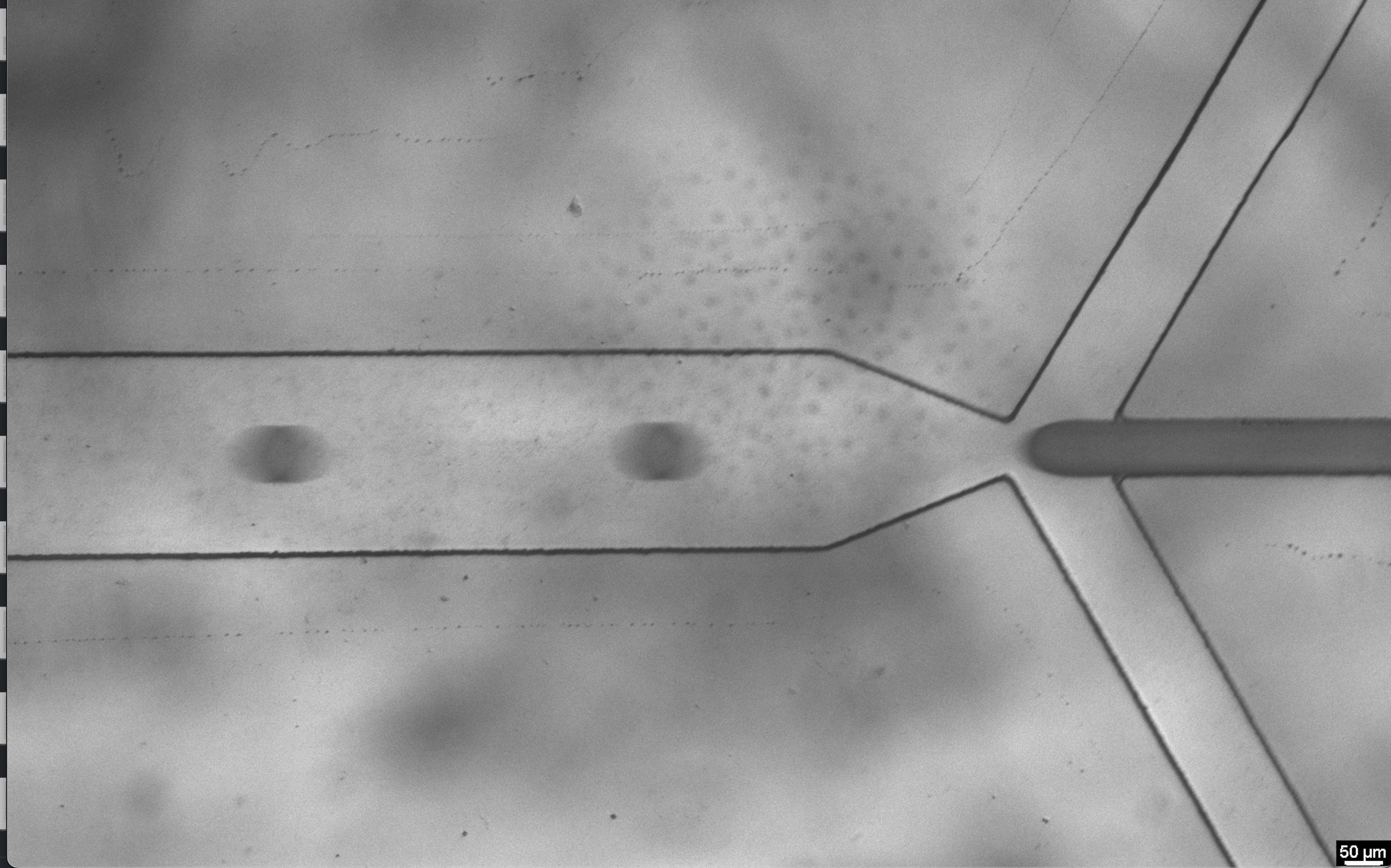


**Figure S3.** Micrograph of the flow-focusing generator making evenly-spaced ferrofluid-in-oil emulsions.


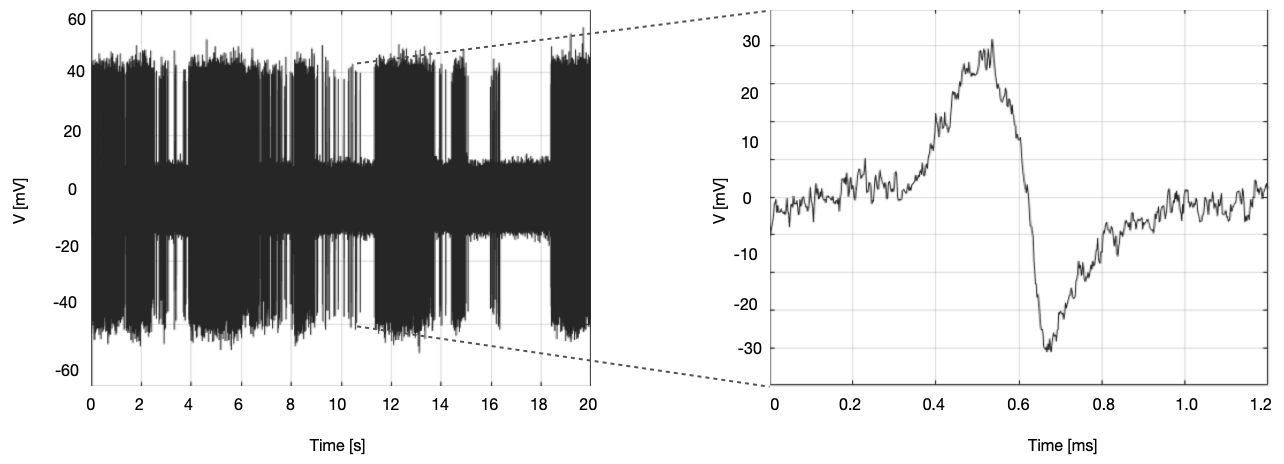


**Figure S4.** Ferrofluid droplet signals over the course of 20 seconds and a zoom-in of the V_H_ from one representative droplet.


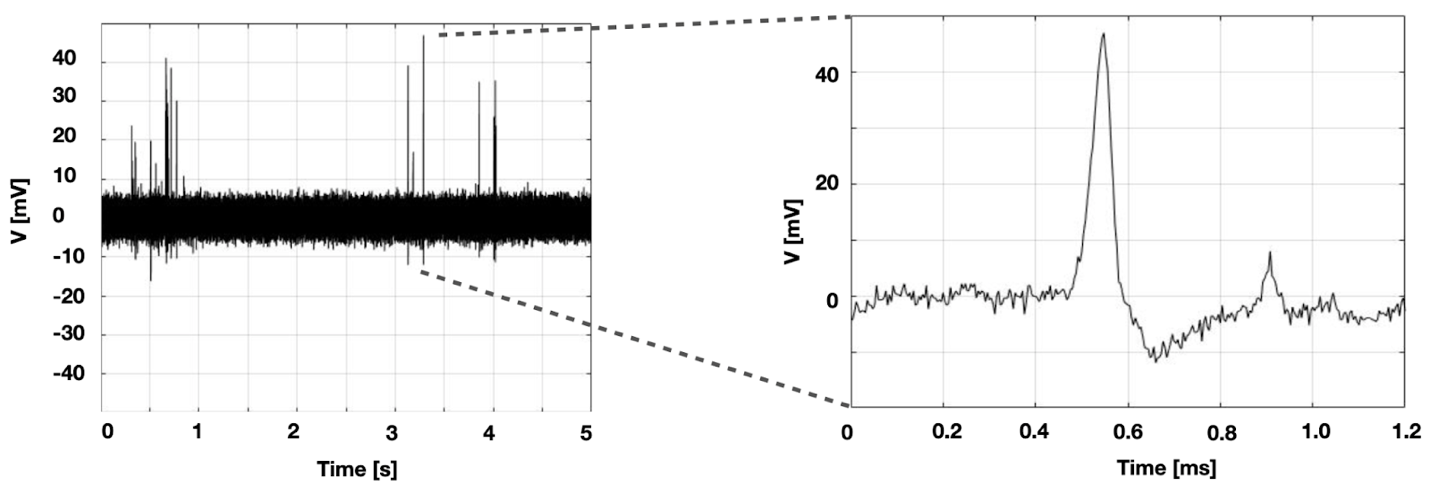


**Figure S5.** Agarose bead signals in BSA over the course of 5 seconds and a zoom-in of the V_H_ from one representative bead.

**
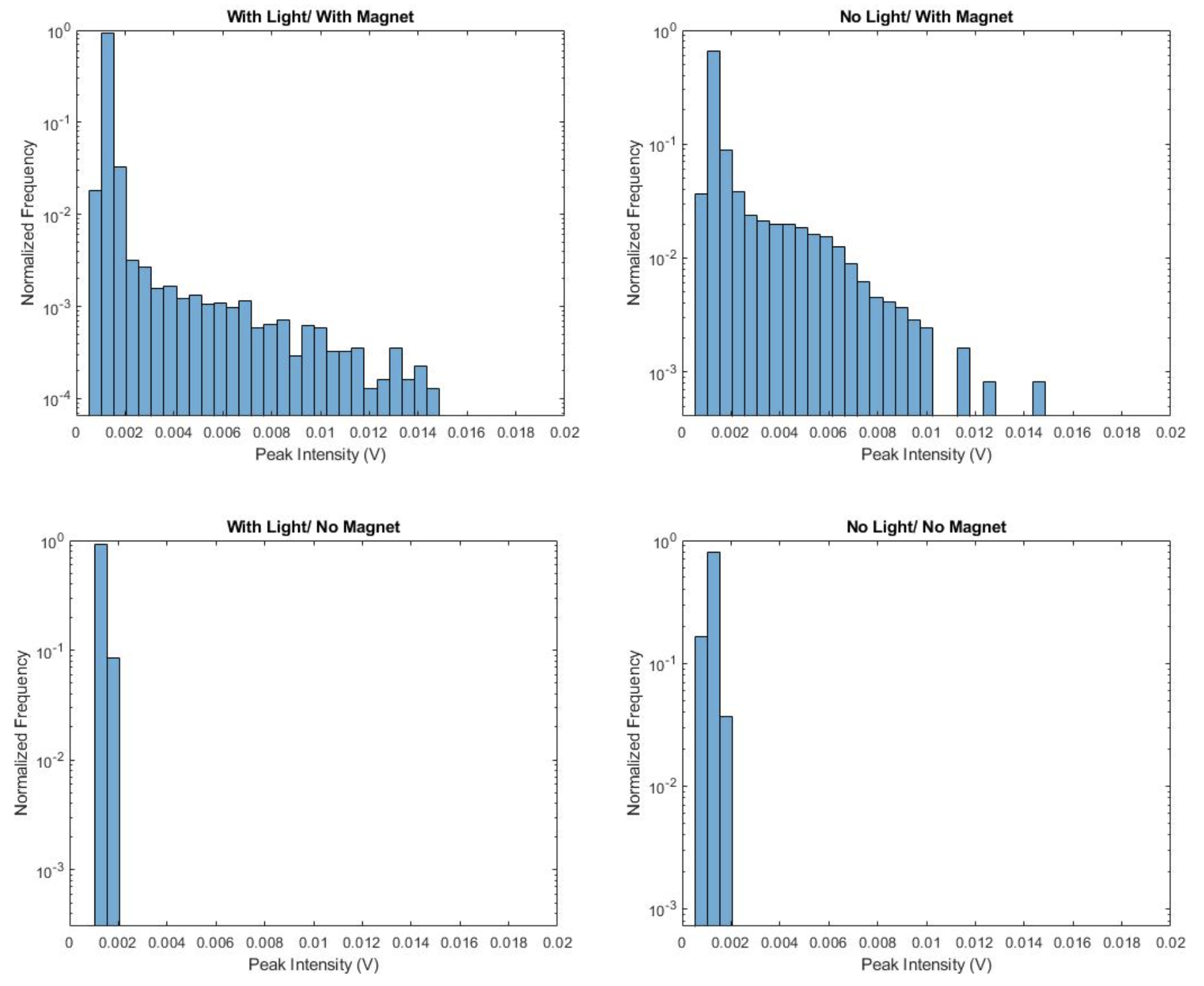
Figure S6.** Histograms of different controls to confirm the signal is a magnetic response while agarose beads are over flowing the sensor. a. With ambient light and with external magnetic field. b. Without ambient light and with external magnetic field. c. With ambient light and without external magnetic field. d. Without ambient light and without external magnetic field.

**a**

**b**

**c**

**d**


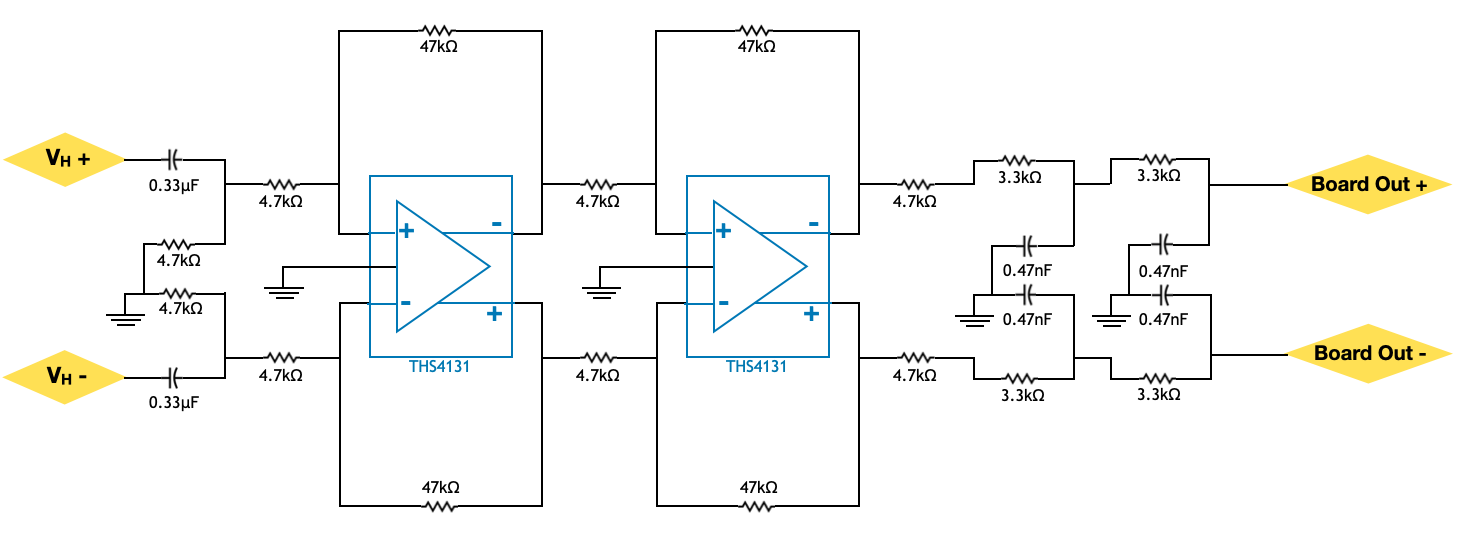


**Figure S7.** Schematic of the amplifier circuit composed of a high-pass filter and two differential amplifiers (10x gain each).
